# A comparison of automated urine analyzers cobas 6500, UN 3000-111b and iRICELL 3000 with manual microscopic urinalysis

**DOI:** 10.1016/j.plabm.2021.e00203

**Published:** 2021-01-18

**Authors:** Piraya Tantisaranon, Kanyarat Dumkengkhachornwong, Peechana Aiadsakun, Areerat Hnoonual

**Affiliations:** a Emergency Laboratory, Department of Pathology, Faculty of Medicine, Prince of Songkla University, Songkhla, Thailand; b Clinical Microscopy Laboratory, Department of Pathology, Faculty of Medicine, Prince of Songkla University, Songkhla, Thailand; c Division of Human Genetics, Department of Pathology, Faculty of Medicine, Prince of Songkla University, Songkhla, Thailand

**Keywords:** Urine sediment, Automated urine analyzer, Microscopic, Urinalysis

## Abstract

**Objectives:**

Microscopic examination is essential in urine analysis. This is a simple way to collect informative data but it is also labor-intensive, time-consuming, and requires experienced staff for accurate results and interpretation. Several automated urine analyzers have been introduced for urine analysis in medical laboratories. The aim of this study was to assess and compare the performance of the most common three automated urine analyzers, Cobas 6500, UN3000-111b and iRICELL 3000.

**Design:**

and Methods: A total of 100 routine urine samples were used in the study. Results from the three machines were compared with the routine procedure results including physical, chemical and sediment analysis.

**Results:**

There was good correlation of urine physical and chemical analyses between the three analyzers with an overall concordance level of more than 80%. For sediment analysis, the degree of concordance between manual analysis and the three instruments was very good to good for white blood cells, red blood cells and epithelial cells, and moderate for bacteria. There were fair to good agreements between manual microscopy and the three instruments, Cobas 6500, UN3000-111b and iRICELL 3000, for casts (Cohen’s kappa 0.42, 0.38 and 0.62, respectively).

**Conclusions:**

The three automated urine analyzers showed similar performances and good correlation with manual microscopy. The results of this study indicate that automated urine analyzers could be used for initial urine testing to reduce high workloads and to save time, but manual microscopic analysis by experienced staff is still necessary to classify urine sediments for confirmation, especially in pathologic specimens.

## 1 Introduction

Urinalysis is an essential screening test in clinical laboratories in order to diagnose and plan treatment for urinary tract infections, kidney disease and diabetes. Urinalysis includes visual examination, evaluation of chemical parameters and microscopic examination. The traditional procedure for urinalysis recommended by the Clinical and Laboratory Standards Institute (CLSI) and the European Urinalysis Guidelines consists of two steps [1,2]. The first step includes visual inspection and a dipstick test, and the second step is microscopic examination of the urine specimen sediment to look for erythrocyturia, leukocyturia, bacteriuria or proteinuria. Although manual microscopic techniques are standardized, the traditional microscopic examination of the urinary sediment is labor intensive, time consuming, imprecise, inaccurate and has wide interobserver variability. Therefore, automatic urine analyzers for high-volume laboratory settings were developed in order to improve the certainty of quality measurements and save labor and time [3]. Several manufacturers have developed automated urine analyzers which integrate and perform all urinalysis components for use in clinical laboratories. There are two main types of analyzers, based on different technologies. The first type is an image-based microscopic urine sediment analysis that uses a video camera to capture and sort particles according to preset particle dimensions, and the second is based on the principle of flow cytometry.

In recent years, a new generation of more sophisticated automated urinalysis machines has been introduced. Each platform has different advantages and disadvantages [[3], [4], [5]]. Automated microscopic and strip analyzers have been combined into fully automated workstations. Of these, the UN 3000-111b (Sysmex Corporation, Kobe, Japan) which uses the flow cytometry method, and the iRICELL 3000 (Iris Diagnostics, Chatsworth, USA) and Cobas 6500 (Roche, Mannheim, Germany), which use an image-based analysis system, were used in our study. The aim of the study was to compare the performance of these three automated urine analyzers with different technologies with manual microscopy, and to compare their chemical and microscopic examination components. The study was undertaken to help us decide which machine to purchase for our laboratory.

## 2 Materials and methods

### 2.1 Urine specimens

One hundred freshly-collected urine specimens from admitted in- and out-patients which were submitted to the Microscopy Laboratory of the Department of Pathology, Faculty of Medicine, Prince of Songkla University, Thailand for routine diagnostic urinalysis were randomly selected and included in the study. The samples were collected in preservative-free containers and transferred to four different test tubes for three automated urine analyzers without centrifuging and manual microscopy by 3 experienced medical technologists, independently using the same microscope slide. All samples were analyzed within 2 ​h of their arrival at the laboratory.

This study was approved by the Institutional Ethics Committee of the Faculty of Medicine, Prince of Songkla University (REC.62-470-5-7).

### 2.2 Automated urine analyzers

#### 2.2.1 Cobas 6500

The Cobas 6500 is a combination of 2 analyzers including the Cobas u601 which analyzes physical and chemical components, and the Cobas u701 which performs microscopic examinations. The Cobas u601 can evaluate leukocytes, erythrocytes, nitrite, protein, glucose, ketones, urobilinogen, bilirubin, pH, colour and turbidity with reflectance photometry and specific gravity with refractometry. For the Cobas u701, a urine specimen is pipetted into a special cuvette and the machine centrifuges the cuvette at 2000 ​rpm for 10 ​s. A microscopic camera takes 15 real images of each centrifuged specimen that are then analyzed by particle recognition software for erythrocytes, leukocytes, squamous epithelial cells, non-squamous epithelial cells, bacteria, hyaline casts, pathological casts, crystals, yeasts, mucus and sperm.

#### 2.2.2 Sysmex UN3000-111b

The UN3000-111b has 2 component analyzers in a single platform including a chemical component (UC-3500 analyzer) for analyzing the physical and chemical parts of urine and a fluorescence flow cytometry component (UF-5000 analyzer) for microscopic examination of sediments. The UC-3500 analyzer can evaluate leukocytes, erythrocytes, nitrite, protein, glucose, ketones, urobilinogen, bilirubin, pH, colour and turbidity using reflectance photometry and specific gravity using refractometry. Particle identification and characterization are achieved by detection of forward scatter and fluorescence, and adaptive cluster analysis is performed with the UF-5000 analyzer. Additionally, the UN3000-111b includes the UD-10 which is a urine particle digital imaging device, capturing images of urine and classifying the particles into 8 classes on the basis of their size, and giving a detailed view of urine particles to the user. The UD-10 device confirms abnormal results after particle analysis.

#### 2.2.3 iRICELL 3000

The iRICELL 3000 is a combination of 2 component analyzers including a chemical component (iChemVELOCITY) and a microscopic examination component (iQ200). The Iris iQ200 uses Digital Flow Morphology technology with Auto-Particle Recognition (APR) software to classify and quantify the cells and particles in uncentrifuged urine. In the system, the particles in a urine specimen are imaged in a planar plane in flow cells by hydrodynamic focusing sheath fluid, then the hundreds of captured images from its digital camera are identified and counted by APR software, and each particle is auto-classified by size, shape, contrast and texture. The results are classified into 12 particle categories, red blood cells (RBCs), white blood cells (WBCs), WBC clumps, hyaline casts, pathological casts (or unclassified casts), squamous epithelial cells, non-squamous epithelial cells, bacteria, yeasts, crystals, mucus and sperm.

The characteristics of the Cobas 6500, UN 3000-111b and iRICELL 3000 are provided in Table 1.

**Table 1:** Specifications of the Cobas 6500, UN 3000-111b and iRICELL 3000.

General Characteristic	Cobas 6500	UN 3000-111b	iRICELL 3000
Specimen application	Pipetted	Pipetted	Pipetted
Sample throughput per hour	240 (Cobas u 601)	276 (UC-3500 Chemistry)	210 (iChemVELOCITY)
	116 (Cobas u 701)	105 (UF-5000 Particles compensation by Fluorescence Flow Cytometry	101 (iQ200 Sprint)
		105 (UF-5000 Particles compensation) by Fluorescence Flow Cytometry	
Specimen consumption	2.3 ​mL	0.98 ​ml	2.2 ​ml
Required specimen volume	2.8 ​mL	2.6 ​ml	3.0 ​ml
Data memory (tests)	10,000	100,000 tests for U-WAM; 10,000 tests for UC3500; 10,000 tests for UC3500; 400 tests for UD10	10,000
Physical dimensions	Width: 179 ​cm;Depth: 53 ​cm; Height: 64.4 ​cm	Width: 192 ​cm; Depth: 90 ​cm; Height: 87 ​cm	Width: 150.5 (including workstation); Depth: 61 ​cm; Height: 56 ​cm
Physical and Chemical Parts			
Turbidity method	Turbidimeter	Turbidity correction performed by Flow Unit	Scattered light
Specific gravity method	Refractometry (automatic temperature compensation)	Refractometry technology	Refractive Index
Wave lengths used	465, 528, 560, 615 ​nm	Test strip photometry principle scanned with a colour CMOS sensor and photometry is performed, creating two-dimensional photometry	472 ​nm, 520 ​nm, 630 ​nm
Microscopic Components			
Specimen application	Cuvette	Flow cytometry system	Planar flow system
Centrifuge	2000 ​rpm for 10 ​s	No	No
Principle of analysis	Digital imaging and particle recognition software	Fluorescence Flow Cytometry	Digital flow morphology using APR software
Stain	No	Yes (fluorescence by UF5000)	No
Image	Monochrome	Monochrome (by UD10)	Monochrome
Output of images	Images available	Images available	Image available

### 2.3 Manual microscopic examinations

Manual microscopic sediment examinations were performed following the CLSI approved guidelines for urinalysis [2]. 10 ​mL of each urine specimen was centrifuged at 1500 ​rpm (325×*g*) for 5 ​min. After centrifugation, the sediment contents were resuspended and then slides were prepared and examined by light microscope at magniﬁcations of 100x (low power field; LPF) for casts and 400x (high-power field; HPF) for RBCs, WBCs, epithelial cells, and bacteria. The particles were counted per field, and the results were classified into 3 categories for evaluation (Supplemental Data Table S1). WBCs, RBCs and epithelial cells were classified semi-quantitatively (0–5, 6–10, 11–20, and >20 ​cells/HPF). Bacteria were classified as negative, few, moderate, or numerous. Casts were classified as not found and found. Manual microscopy was used as the reference standard method for urine sediment evaluation in all reports.

All samples were independently examined by 3 experienced medical technologists using the same microscope slide. The results were accepted when two or three evaluators reported the same category of cells or particles. If all 3 assessors reached notably different results for any particular slide, the analysis was repeated with a new urine sample in order to resolve the discrepancy.

### 2.4 Evaluation protocols and analysis of results

#### 2.4.1 Precision test

In order to evaluate the analytical performances of the workstations, between- and within-run variations and carry-over measurements of the workstations were evaluated against control materials. Liquichek, urinalysis control level 1 and 2 (Biorad Laboratories, CA, USA) was used to provide analytical quality control, including for WBCs and RBCs. We used 20 repetitions for both within-run (20 times within a day) and between-run (once per day on 20 separate days) precision. The precision of each automated urine analyzer was assessed by mean ​± ​SD and percentage coefficient of variation (CV%). CV values less than 30% are considered to be acceptable.

#### 2.4.2 Physical and chemical components

We compared the specific gravity results of the three automated urine analyzers with refractometry and compared the results statistically using regression analysis and coefficient correlation (*r*). For the chemical components, the analytical detection of pH, glucose, protein, bilirubin, urobilinogen, blood, ketones, nitrite, and leukocyte esterase was compared between the analyzers. The analytical assessment of glucose and protein between the three machines was compared with the results of a Cobas c501 analyzer, a fully automated analyzer, which has been wildly used for clinical chemistry testing and has been used in our laboratory for a long time. The Cobas c501 performs urinalysis based on standard methods, notably colorimetry for protein analysis and an enzymatic method for glucose analysis. The results of the analysis of the chemical components of the three machines were considered to be concordant if they were within one grading difference result from the Cobas c501. The pairwise concordance rates between the three instruments were defined as the percentage of results within ±1 grade from the best-fit line.

#### 2.4.3 Urine sediment comparisons

For the sediment component of the study, we evaluated WBCs, RBCs, epithelial cells, and casts, comparing between the automated and the manual method for each analyzer, and among the automated analyzers. Cohen’s kappa coefficient was used to estimate agreements between the manual method and the automated urine analyzer results. Values for kappa coefficient of 0–0.20, 0.21–0.40, 0.41–0.60, 0.61–0.80 and 0.81–1.00 were characterized as poor, fair, moderate, good, and very good agreement, respectively. We categorized the data for casts as not found or found. The sensitivity, specificity, positive predictive value (PPV) and negative predictive value (NPV) were calculated for these data. Concordance rates of urine sediment with the same grade and within ±1 grade difference between the machines were calculated.

## 3 Results

### 3.1 Precision test

The within-run and between-run coefficients of variations of the RBCs and WBCs for the three automated urine analyzers, Cobas 6500, UN3000-111b, and iRICELL 3000, are shown in Table 2. The CV% from the within-run and between-run precision of the level 1 control for Cobas 6500 was lower than the other two analyzers. However, within-run and between-run precision of the three automated urine analyzers for RBCs and WBCs were acceptable at all levels (CV ​< ​30%).

**Table 2:** Coefficients of variation of microscopic analysis for red blood cells and white blood cells from Cobas 6500, UN3000-111b and iRICELL 3000.

Analyzer	Parameter	Within-run imprecision				Between-run imprecision			
		Level 1 (Low)		Level 2 (High)		Level 1 (Low)		Level 2 (High)	
		mean ​± ​SD (Cell/μl)	CV (%)	mean ​± ​SD (Cell/μl)	CV (%)	mean ​± ​SD (Cell/μl)	CV (%)	mean ​± ​SD (Cell/μl)	CV (%)
Cobas 6500	RBCs	0.0^a^	0.0^a^	186.26 ​± ​8.26	4.43	0.0^a^	0.0^a^	190.29 ​± ​7.70	4.05
	WBCs	0.0^a^	0.0^a^	26.95 ​± ​1.55	5.77	0.0^a^	0.0^a^	26.68 ​± ​1.65	6.18
UN3000-111b	RBCs	0.216 ​± ​0.04	17.36	112.25 ​± ​2.06	1.83	0.2 ​± ​0.08	43.93	113.14 ​± ​1.66	1.46
	WBCs	0.0^a^	0.0^a^	0.45 ​± ​0.10	22.44	0.0^a^	0.0^a^	0.44 ​± ​0.10	23.91
iRICELL 3000	RBCs	3.60 ​± ​0.50	13.96	76.70 ​± ​3.23	4.21	4.6 ​± ​1.19	26.18	78.35 ​± ​4.22	5.39
	WBCs	0.0^a^	0.0^a^	26.10 ​± ​1.52	5.82	0.0^a^	0.0^a^	24.75 ​± ​2.40	9.71

a Indicates that SD and percent of coefficient of variance (CV) could not be calculated because the mean value of RBC and WBC results was 0 ​cells per μl. RBCs: red blood cells; WBCs: white blood cells; SD: standard deviation.

### 3.2 Chemical components

**Fig. 1:**
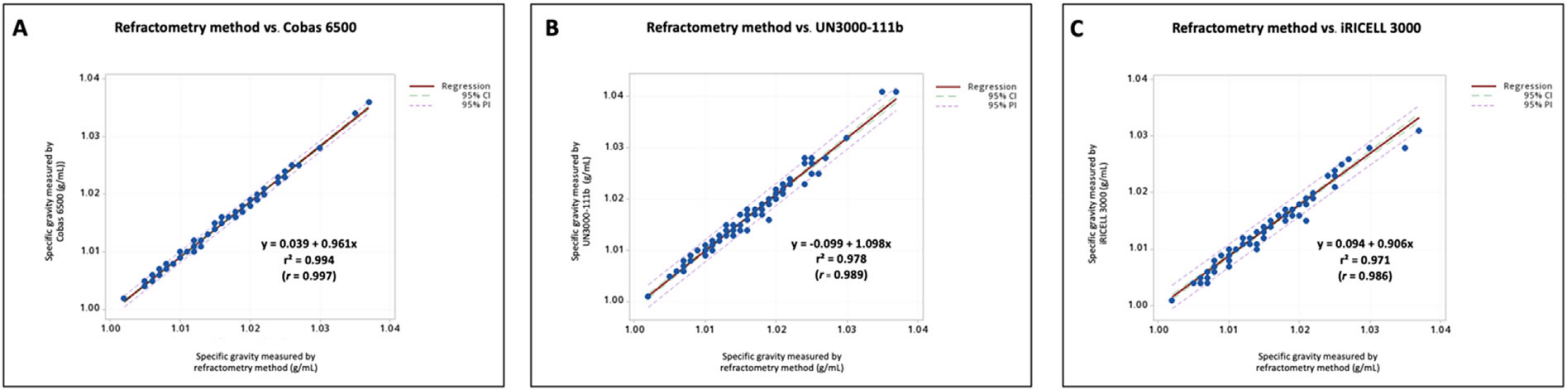
Comparison of the refractometry method with the three urine analyzers for specific gravity. Correlation between refractometry and Cobas 6500 (*r* ​= ​0.99) **(A)**. Correlation between refractometry and UN3000-111b (*r* ​= ​0.99) **(B)**. Correlation between refractometry and iRICELL 3000 (*r* ​= ​0.99) **(C)**. All of these correlation points were statistically significant (*P* ​< ​0.01). r^2^: Coefficient of determination; r: Pearson correlation coefficient; CI: confidence interval; PI: prediction interval.

Pearson’s coefficient correlation (*r*) and linear regression analysis of specific gravity for comparison of the machine results with the refractometry method results as the gold standard method are presented in Fig. 1.

A pairwise table of the chemical components which demonstrates the concordance of the strip test results within the same grading rate and within one grading difference is summarized in Table 3. The strip tests of the three automated urine analyzers, Cobas 6500 (cobas u601), UN3000-111b (UC-3500) and iRICELL 3000 (iChemVELOCITY), showed a concordance level of more than 80% within one grading difference of all parameter determinations. The best concordance level for all analyzers was obtained for nitrite (100%), while the poorest results were for bilirubin (88–92%) and leukocyte esterase (84–98%). The concordances in protein determination between the Cobas c501 and the Cobas 6500, UN3000-111b, and iRICELL 3000 were 100, 93 and 95, respectively, while the concordances in glucose determination between the Cobas c501 and the Cobas 6500, UN3000-111b, and iRICELL 3000 were 94, 96 and 96, respectively (data not shown).

**Table 3:** Correlations represented as concordance rate (%) and concordance rate within ±1 grading difference (%) of the chemistry analysis results between the automated urine analyzers, Cobas 6500 (cobas u601), UN3000-111b (UC-3500) and iRICELL 3000 (iChemVelocity).

Parameter	Cobas 6500 vs UN3000-111b		Cobas 6500 vs UN3000-111b		iRICELL 3000 vs UN3000-111b	
	Concordance rate (%)	Concordance rate within ±1 grading difference (%)	Concordance rate (%)	Concordance rate within ±1 grading difference (%)	Concordance rate (%)	Concordance rate within ±1 grading difference (%)
pH	44	98	60	88	46	100
Glucose	96	98	96	96	98	100
Protein	58	98	64	98	82	100
Bilirubin	92	92	88	88	92	92
Urobilinogen	94	96	96	98	96	96
Blood	68	98	64	90	80	100
Ketones	92	94	92	94	100	100
Nitrite	96	100	96	100	100	100
Leukocyte esterase	78	92	54	84	70	98

### 3.3 Sediment comparison

**Fig. 2:**
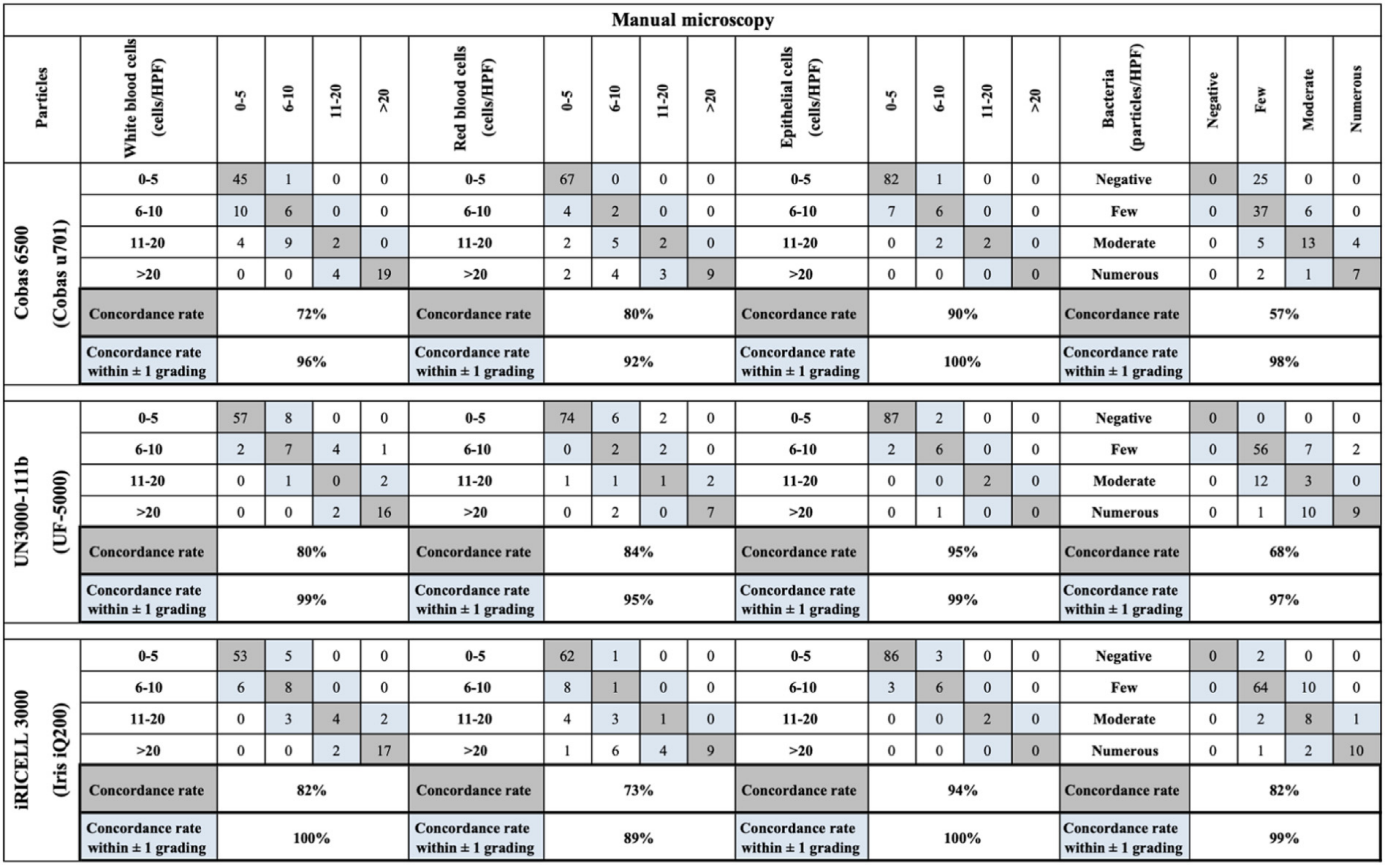
Comparison of pairwise urine sediment results among the three instruments and manual microscopy. The gray-shaded areas represent the number of cases within the same grade and the blue-shaded areas represent the number of cases within one grade difference. HPF: high-power field. (For interpretation of the references to colour in this figure legend, the reader is referred to the Web version of this article.)

The pairwise agreements within the same grade and within one grade difference between the manual and automated methods for RBCs, WBCs, epithelial cells, bacteria and casts are shown in Fig. 2. The best concordance between methods was in the epithelial cell counts and the least concordance between methods was in the bacteria counts. Agreement within the same grade between the Cobas 6500 (Cobas U701) and the manual method was lower than the agreement between the UN3000-111b (UF-5000), iRICELL 3000 (Iris IQ200) and the manual method for WBCs, epithelial cells and bacteria. The pairwise agreements between the automated urine analyzers are shown in Supplemental Data Table S2.

The evaluations of concordance of urine sediments between the 3 machines are shown in Table 4 and Supplemental Data Table S3. There was good agreement between the manual method and the automated analyzers for RBCs, WBCs, epithelial cells and bacteria. For WBCs, RBCs and epithelial cells, the concordances within the same grade difference between manual microscopy and automated urine analyzers had very good to good agreement. For bacteria, all concordances between manual microscopy and automated urine analyzers had moderate agreement. For casts, the concordance between manual microscopy and the iRICELL 3000 (Iris IQ200) was much better than the concordance between manual microscopy and the other two instruments.

**Table 4:** Degrees of concordance represented as weighted Cohen’s kappa results between the manual method and the three analyzers.

	Weighted kappa results (95% CI)		
	Manual vs Cobas 6500	Manual vs UN3000-111b	Manual vs iRICELL 3000
White blood cells	0.75 (0.66–0.84)^c^	0.81 (0.74–0.89)^d^	0.85 (0.78–0.92)^d^
Red blood cells	0.70 (0.57–0.82)^c^	0.72 (0.58–0.85)^c^	0.62 (0.50–0.74)^c^
Epithelial cells	0.67 (0.48–0.86)^c^	0.76 (0.57–0.96)^c^	0.74 (0.53–0.95)^c^
Bacteria	0.48 (0.36–0.60)^b^	0.51 (0.36–0.65)^b^	0.45 (0.27–0.63)^b^
Casts	0.42 (0.17–0.67)^b^	0.38 (0.10–0.66)^a^	0.62 (0.38–0.86)^c^

a Fair agreement (kappa between 0.21 and 0.40).

b Moderate agreement (kappa between 0.41 and 0.60).

c Good agreement (kappa between 0.61 and 0.80).

d Very good agreement (kappa between 0.81 and 1.00). CI: confidence interval.

Analytical sensitivity, specificity, and positive and negative predictive value were assessed relative to the manual microscopic results (Table 5). For WBCs, RBCs, epithelial cells and bacteria, the Cobas 6500 (Cobas u701), UN3000-111b (UF-5000), and iRICELL 3000 (Iris IQ200) showed relatively high both sensitivity and specificity, while the specificity of casts in the automated instruments was found to be better than the sensitivity.

**Table 5:** Sensitivity, specificity and predictive values of sediment analysis for the automatic urine analyzers compared with manual microscopy.

	Sensitivity (%)	Specificity (%)	Positive predictive value (%)	Negative predictive value (%)
**White blood cells**				
Cobas 6500	97.6	76.3	74.1	97.8
UN3000-111b	80.5	96.6	94.3	87.7
iRICELL 3000	87.8	89.8	85.7	91.4
**Red blood cells**				
Cobas 6500	100.0	89.3	75.8	100.0
UN3000-111b	68.0	98.7	94.4	90.2
iRICELL 3000	96.0	82.7	64.9	98.4
**Epithelial cells**				
Cobas 6500	90.0	92.1	58.8	98.8
UN3000-111b	81.8	97.8	81.8	97.8
iRICELL 3000	72.7	96.6	72.7	96.6
**Bacteria**				
Cobas 6500	75.0	Undetemined^a^	100.0	Undetemined^a^
UN3000-111b	100.0	Undetemined^a^	100.0	Undetemined^a^
iRICELL 3000	98.0	Undetemined^a^	100.0	Undetemined^a^
**Casts**				
Cobas 6500	50.0	91.9	91.9	50.0
UN3000-111b	28.7	98.8	80.0	89.5
iRICELL 3000	57.1	97.7	80.0	93.3

a No samples of true negatives or false positives were available for calculation.

## 4 Discussion

In recent years, several automated urine analyzers have been introduced for use in medical laboratories. To our knowledge, this is the first study to compare the performance of three of the most popular of these fully automated urine analyzers, the Cobas 6500, UN3000-111b and iRICELL 3000 in terms of their physical, chemical and sediment analysis performances. For the physical and chemical analyses, the specific gravity measurements of the three instruments had excellent correlation with the standard laboratory refractometry. The strip test results of the three automated analyzers were very similar for all chemical parameters.

For urine sediment, the between-run and within-run precision of the three analyzers for WBCs and RBCs were lower for the specimens with fewer cells (control level 1). Although, within-run and between-run precision of the three automated urine analyzers for RBCs and WBCs were acceptable at all levels, only the Cobas 6500 showed a high degree of precision of both RBCs and WBCs with CV < 10%. The precision of the data which was produced by the three analyzers in this study was similar to previous studies [6,7]. The concordance between the manual method and the three instruments was very good to good for WBCs, RBCs and epithelial cells, while being only moderate for bacteria. Similar to other studies, most of the problems with the automated urine analyzers occurred in bacteria analysis, and studies have suggested that the presence of bacteria in urine samples should be confirmed by manual microscopy [[8], [9], [10]]. In this study, the three automated instruments demonstrated good diagnostic sensitivity and specificity for WBCs, RBCs and epithelial cells, while the specificity of the automated instruments for casts was higher than their sensitivity in all three instruments. The detection of casts by automated systems is difficult. We found that the concordance between the manual method and the instruments showed fair to good agreement with the manual method for casts, a finding which was similar to other studies [8,11,12]. Therefore, it is necessary to confirm the presence of casts in urine samples by manual microscopy. The negative predictive values of the three analyzers in our study were similar and better than the positive predictive values, indicating that these three analyzers have low false-negative results but higher false-positive results. Therefore, if using these machines, the technician should review sediment findings, especially in pathological urine testing.

Several microscopy comparison studies have shown that automated analyzers mostly have similar performances and with results comparable with manual microscopy. One of these studies, conducted by Baken et al. [13], compared the fully automated urine sediment analyzers Cobas 6500 and Iris IQ200 with manual microscopy, and found that the two analyzers showed similar performances and good compatibility to manual microscopy for WBCs, RBCs and squamous epithelial cells. Another study by Baken et al. [14] compared the Cobas 6500 and the Sysmex UN series of aumated analyzers with manual microscopy for sediment components. These automated urine analyzers showed good concordance and the comparison with manual microscopy showed that the automated sediment analyzers had satisfactory analytical performances for formed elements. Akin et al. [6] compared two fully automated urine analyzers (the UriSed and iQ200), and the results showed statistically significant correlations among the manual, UriSed and iQ200 methods. İnce et al. [8] reported that the iQ200 and Dirui FUS-200 had comparable results with each other and with manual microscopy for WBCs, RBCs and epithelial cells. Another study compared the diagnostic performance of three automated urinalysis systems, the Iris IQ200, Sysmex UF-1000i and UriSed LabUMat, and found acceptable correlations in chemical data analysis between the instruments [11]. In addition, good correlation of the physical, chemical and sediment parts of urine have been reported in studies comparing the UX-2000 and Cobas 6500 machines [7]. Although all of these studies reported similar good performance for chemical and sediment examination between automated urine analyzers and manual microscopy, and between instruments, confirmation of pathological results from automated systems by manual examination is still recommended [7].

The limitations of the present study were the small sample size and the low number of pathological samples, thus further studies are needed with larger sample sizes to validate our findings. Despite these limitations, this study has the benefit that the measurements were obtained from real patient samples and therefore the results can be applied directly to the clinical situation.

## 5 Conclusions

The Cobas 6500, UN3000-111b and iRICELL 3000 analyzers showed similar performances in physical, chemical and sediment examinations in most of the parameters analyzed. Therefore, we can conclude that the these three fully automated urine analyzers can be safely used in urine analysis. The automated urine analyzers can reduce the workload involved in analysis of a large number of urine samples, save time, and decrease human error in preparations for microscopic analysis, such as centrifugation. However, manual urinalysis is still recommended for confirmation of findings, particularly in urine samples flagged as some type of pathology.

## Funding

This work was supported by three companies – 10.13039/100016545Roche Diagnostics (Mannheim, Germany), Sysmex Corporation (Kobe, Japan), and Iris Diagnostics (Chatsworth, USA).

## CRediT authorship contribution statement

Piraya Tantisaranon: Conceptualization, Methodology, Validation, Data curation, Formal analysis, Writing - original draft. Kanyarat Dumkengkhachornwonga: Validation, Investigation. Peechana Aiadsakunb: Validation, Investigation. Areerat Hnoonual: Conceptualization, Writing - review & editing, Supervision.

## Declaration of competing interest

None.
