# Supplementary material for: A comparison of automated urine analyzers cobas 6500, UN 3000-111b and iRICELL 3000 with manual microscopic urinalysis
Source: Pract Lab Med. 2021 Jan 18;24:e00203. doi: 10.1016/j.plabm.2021.e00203 (PMC7847969; doi:10.1016/j.plabm.2021.e00203)
Supplement: Multimedia component 1 [file mmc1.pdf]

Supplemental Data Table S1. Semi-quantitative range classification of urine sediment

Parameters	Ranges			
**White blood cells (cells/HPF)**	0-5	6-10	11-20	>20
**Red blood cells (cells/HPF)**	0-5	6-10	11-20	>20
**Epithelial cells (cells/HPF)**	0-5	6-10	11-20	>20
**Bacteria (cells/HPF)**	Negative	Few	Moderate	Numerous
**Casts (cells/LPF)**	Not found	Found		

Abbreviations: HPF, high-power field; LPF, low-power field

Supplemental Data Table S2. Comparison of pairwise urine sediment results between the automated urine analyzers. The gray-shaded areas represent number of cases within the same grade and blue-shaded areas represent number of cases within one grade difference.

	Cobas 6500 (Cobas u701)																			
Particles	White blood cells (cells/HPF)	0-5	6-10	11-20	>20	Red blood cells (cells/HPF)	0-5	6-10	11-20	>20	Epithelial cells (cells/HPF)	0-5	6-10	11-20	>20	Bacteria (particles/HPF)	Negative	Few	Moderate	Numerous
UN3000-111b (UF-5000)	0-5	46	12	7	0	0-5	67	4	7	4	0-5	82	7	0	0	Negative	0	0	0	0
	6-10	0	4	7	3	6-10	0	0	2	2	6-10	1	6	1	0	Few	25	31	6	3
	11-20	0	0	1	2	11-20	0	1	0	4	11-20	0	0	2	0	Moderate	0	12	3	0
	>20	0	0	0	18	>20	0	1	0	8	>20	0	0	1	0	Numerous	0	0	13	7
	Concordance rate	69%				Concordance rate	75%				Concordance rate	90%				Concordance rate	41%			
	Concordance rate within ± 1 grading	90%				Concordance rate within ± 1 grading	86%				Concordance rate within ± 1 grading	100%				Concordance rate within ± 1 grading	97%			

	Cobas 6500 (Cobas u701)																			
Particles	White blood cells (cells/HPF)	0-5	6-10	11-20	>20	Red blood cells (cells/HPF)	0-5	6-10	11-20	>20	Epithelial cells (cells/HPF)	0-5	6-10	11-20	>20	Bacteria (particles/HPF)	Negative	Few	Moderate	Numerous
iRICELL 3000 (Iris iQ200)	0-5	43	11	4	0	0-5	59	3	0	1	0-5	83	6	0	0	Negative	2	0	0	0
	6-10	3	5	6	0	6-10	7	0	2	0	6-10	0	7	2	0	Few	23	39	12	0
	11-20	0	0	5	4	11-20	1	2	5	0	11-20	0	0	2	0	Moderate	0	4	6	1
	>20	0	0	0	19	>20	0	1	2	17	>20	0	0	0	0	Numerous	0	0	4	9
	Concordance rate	72%				Concordance rate	81%				Concordance rate	92%				Concordance rate	56%			
	Concordance rate within ± 1 grading	96%				Concordance rate within ± 1 grading	97%				Concordance rate within ± 1 grading	100%				Concordance rate within ± 1 grading	100%			

	iRICELL 3000 (Iris iQ200)																			
Particles	White blood cells (cells/HPF)	0-5	6-10	11-20	>20	Red blood cells (cells/HPF)	0-5	6-10	11-20	>20	Epithelial cells (cells/HPF)	0-5	6-10	11-20	>20	Bacteria (particles/HPF)	Negative	Few	Moderate	Numerous
UN3000-111b (UF-5000)	0-5	54	11	0	0	0-5	62	9	7	4	0-5	87	2	0	0	Negative	0	0	0	0
	6-10	4	3	7	0	6-10	0	0	1	3	6-10	2	6	0	0	Few	2	57	3	3
	11-20	0	0	1	2	11-20	1	0	0	4	11-20	0	0	2	0	Moderate	0	13	2	0
	>20	0	0	1	17	>20	0	0	0	9	>20	0	1	0	0	Numerous	0	4	6	10
	Concordance rate	75%				Concordance rate	71%				Concordance rate	95%				Concordance rate	69%			
	Concordance rate within ± 1 grading	100%				Concordance rate within ± 1 grading	85%				Concordance rate within ± 1 grading	99%				Concordance rate within ± 1 grading	93%			

Abbreviation: HPF, high-power field.

Supplemental Data Table S3. Degree of concordance represented as weighted Cohen's kappa (%) between the automated urine analyzers.

	Weight kappa (95% CI)		
	**Cobas 6500 vs UN3000-111b**	**Cobas 6500 vs iRICELL 3000**	**iRICELL 3000 vs UN3000-111b**
**White blood cells**	0.68 (0.57-0.78)[c]	0.75 (0.66-0.84)[c]	0.73 (0.70-0.86)[c]
**Red blood cells**	0.55 (0.40-0.70)[b]	0.80 (0.71-0.89)[c]	0.53 (0.37-0.68)[b]
**Epithelial cells**	0.69 (0.51-0.86)[c]	0.73 (0.57-0.90)[c]	0.76 (0.570.96)[c]
**Bacteria**	0.35 (0.24-0.46)[a]	0.49 (0.37-0.62)[b]	0.47 (0.31-0.63)[b]
**Casts**	0.26 (0.00-0.53)[a]	0.53 (0.27-0.78)[b]	0.21 (0.00-0.52)[a]

[a] Fair agreement (kappa between 0.21 and 0.40); [b] Moderate agreement (kappa between 0.41 and 0.60); [c] Good agreement (kappa between 0.61 and 0.80); [d] Very good agreement (kappa between 0.81 and 1.00). CI: confidence interval.
